# Enlargement of the Tonsils—IV

**Published:** 1908-11-14

**Authors:** 


### Laryngology and Rhinology.

### ENLARGEMENT OF THE TONSILS—IV.

### MORCELLEMENT TREATMENT (*continued.*)

The "morcellement" operation is suitable for small irregular tonsils which require removal for recurring inflammation. The operation is done under local anæsthesia, and consists simply in removing as much tonsillar tissue as possible at each sitting with punch-forceps. There are several instruments designed for the purpose; perhaps the best of them is Foxcroft's, the long axis of which corresponds to the axis of the tonsil: it cuts horizontally and is easier to engage than a vertically cutting punch. At least two or three sittings are necessary, and though the operation itself may be made almost painless by careful local anæsthetisation, the resulting soreness is as marked after each sitting as after complete removal. The operation is therefore only to be recommended when the tonsil is small, or when general anæsthesia is refused in a case unsuitable for the guillotine.

### Hæmorrhage after Operation.

Bleeding after removal of the tonsils is always brisk, but usually ceases spontaneously in a few minutes. Apart from cases of hæmophilia, dangerous hæmorrhage is exceedingly rare, but the possibility must never be lost sight of. The bleeding may be primary, or it may recur after an interval, usually of a few hours. Severe hæmorrhage nearly always occurs in adults and in those cases in which the tonsil is fibrosed and cicatricial tissue prevents the proper retraction of the arteries. The bleeding vessel is nearly always one of the arteries within the tonsil; indeed, it is obvious that this must be so if the pharyngeal aponeurosis has not been divided, an accident which is hardly possible with the guillotine or when enucleation is performed by the method described above. As a matter of fact, a large proportion of the severest cases have occurred when the tonsil has been removed with the bistoury and the aponeurosis has probably been cut. The internal carotid is even then practically out of reach, and the injured vessel is probably the facial or ascending pharyngeal, or one of their branches.

### Treatment of Hæmorrhage.

The ordinary brisk hæmorrhage need cause no alarm; its cessation is hastened by freely sponging the face with iced water. If it continue or recur, the first thing is to examine the throat for a tag of tonsil or adenoid accidentally left behind; this is apt to cause continual retching, and may keep up the most severe bleeding, which ceases immediately on removal of the cause. If there be no tag, examine the bed of the tonsil in a good light, wipe away the clot with a sponge, and look for a spurting vessel; if one be found, as is often the case, an artery clip should be applied and left on for several hours. If this cannot be done, or if the bleeding be general, make a firm mass of a mixture of one part of gallic acid to three parts of tannic acid with a very little water, take a piece the size of a walnut, apply it to the surface, and press it firmly on with the finger, making counter-pressure behind the angle of the jaw; this must be maintained as long as possible, several assistants being necessary to relieve each other; a special clamp may be used for the purpose, but is not so well borne and is liable to slip. Other expedients are to apply the cautery at a dull red heat, or to constrict the stump by means of suture passed backwards and forwards through both faucial pillars. In extremely rare cases it may be necessary to ligature arteries in the neck; if this has to be done, the external carotid should be tied and also the ascending pharyngeal and the facial near their origins. Ligature of the internal carotid is useless.

